# Immunoreactivity of Pluripotent Markers SSEA-5 and L1CAM in Human Tumors, Teratomas, and Induced Pluripotent Stem Cells

**DOI:** 10.1155/2013/960862

**Published:** 2013-05-27

**Authors:** Linda Cassidy, Meerim Choi, Jason Meyer, Rui Chang, Gail M. Seigel

**Affiliations:** ^1^Center for Hearing and Deafness, University at Buffalo, 3435 Main Street, Cary 137 Buffalo, NY 14214, USA; ^2^Indiana University, Department of Biology, Indianapolis, IN 46202, USA; ^3^Mount Sinai, Genetics and Genomic Sciences, New York, NY 10029, USA

## Abstract

Pluripotent stem cell markers can be useful for diagnostic evaluation of human tumors. The novel pluripotent marker stage-specific embryonic antigen-5 (SSEA-5) is expressed in undifferentiated human induced pluripotent cells (iPSCs), but little is known about SSEA-5 expression in other primitive tissues (e.g., human tumors). We evaluated SSEA-5 immunoreactivity patterns in human tumors, cell lines, teratomas, and iPS cells together with another pluripotent cell surface marker L1 cell adhesion molecule (L1CAM). We tested two hypotheses: (1) SSEA-5 and L1CAM would be immunoreactive and colocalized in human tumors; (2) SSEA-5 and L1CAM immunoreactivity would persist in iPSCs following retinal differentiating treatment. SSEA-5 immunofluorescence was most pronounced in primitive tumors, such as embryonal carcinoma. In tumor cell lines, SSEA-5 was highly immunoreactive in Capan-1 cells, while L1CAM was highly immunoreactive in U87MG cells. SSEA-5 and L1CAM showed colocalization in undifferentiated iPSCs, with immunopositive iPSCs remaining after 20 days of retinal differentiating treatment. This is the first demonstration of SSEA-5 immunoreactivity in human tumors and the first indication of SSEA-5 and L1CAM colocalization. SSEA-5 and L1CAM warrant further investigation as potentially useful tumor markers for histological evaluation or as markers to monitor the presence of undifferentiated cells in iPSC populations prior to therapeutic use.

## 1. Introduction

Markers associated with pluripotency and stem cell phenotype are often expressed in malignant tissues [1, 2]. These pluripotent markers can serve as useful diagnostic tools [3, 4] or as potential targets for cancer stem cell-directed therapies [5].

Stage-specific embryonic antigen (SSEA) 5 is a novel cell surface marker of pluripotency [6]. Antibodies against SSEA-5 can partially immunodeplete teratoma-forming cells from differentiating iPSC populations [6]. Although SSEA-5 immunoreactivity has been established for iPSCs [6], it is unknown whether SSEA-5 is also expressed in human malignancies. For comparison with SSEA-5, we chose L1CAM, a cell surface adhesion molecule involved in self-renewal and pluripotency of embryonic stem cells [7]. shRNA targeting of L1CAM expression in glioma cells inhibits tumor growth *in vivo* and increases the survival time of tumor-bearing animals [8]. Since both SSEA-5 and L1CAM are cell surface markers of pluripotency, they may also be useful together in monitoring the presence of pluripotent cells in iPSC populations that undergo differentiation. Therefore, we examined human tumors, tumor cell lines, teratomas, and iPSCs for immunoreactivity to SSEA-5 and L1CAM. The goals of this study were to (1) evaluate immunoreactivity of SSEA-5 in human tumors, (2) assess potential colocalization of SSEA-5 with another cell surface marker of pluripotency (L1CAM), and (3) to monitor cell populations for the presence of residual undifferentiated cells in iPSCs that undergo retinal differentiation.

## 2. Materials and Methods

### 2.1. Cell Culture

The IMR90-4 iPS cell line was maintained in mTeSR1 medium (Stem Cell Technologies, Vancouver, CA) on Matrigel (BD Biosciences, San Jose, CA) and differentiated according to published protocols [9]. Retinoblastoma cell line RB143 was grown in RPMI 1640 (Life Technologies, Grand Island, NY) with 10% calf serum (HyClone Laboratories, Inc., Logan, Utah), and prostate carcinoma cell line LNCap Clone FGC cells were grown in RPMI 1640 with 10% fetal bovine serum (American Type Culture Collection ATCC, Manassas, VA). U87-MG and T98G, glioblastoma cell lines, were maintained in EMEM (ATCC) with 10% fetal bovine serum. HCT 116, a colorectal carcinoma cell line, and HT-29, a colon adenocarcinoma cell line, were maintained in McCoy's 5A medium (ATCC) with 10% fetal bovine serum. Capan-1 pancreatic adenocarcinoma cells were cultured in Iscove's Modified Dulbecco's medium (ATCC) with 20% fetal bovine serum. PC-3 prostate adenocarcinoma cells were grown in F-12K medium (ATCC) with 10% fetal bovine serum. Panc-1, a pancreatic carcinoma cell line, was grown in DMEM (ATCC) with 10% fetal bovine serum. Retinoblastoma cell lines Weri-Rb27 (a generous gift from Dr. John Ludlow) and Y79 (ATCC) were cultured in DMEM with 10% calf serum. The cells were maintained in a 37°C incubator with 5% CO_2_. 

MDA-MB-231, a human breast adenocarcinoma cell line, was grown in Leibovitz's L15 medium (ATCC) with 10% fetal bovine serum at 37°C in 100% room air. 

### 2.2. Microarray of ABCG2+/− RB143 Cells

RB143 human retinoblastoma cells were magnetically separated using ABCG2-FITC antibody (Stem Cell Technologies, Vancouver, British Columbia). We chose these two cell populations (ABCG2+ (stem-like cells) versus ABCG2− cells in retinoblastoma) based on our previous studies [10–12]. The resulting ABCG2+ and ABCG2− cells were compared using a microarray (Agilent Technologies), and a list of genes with differential expression was generated. L1CAM was selected from the list for this study based on differential expression, relevance to stem cell biology and cell surface localization for comparison with SSEA-5.

### 2.3. Immunocytochemistry

Cells were rinsed with PBS, spun onto slides in cytospin solution: 72% isopropanol, 19% acetone, and 7.6% glycerol using a Shandon Cytospin II and allowed to dry. 

For diaminobenzidine immunostaining, the Max Poly One kit (Syd Labs, Malden, MA) was used. Slides were blocked for 10 minutes with 3% H_2_O_2_, followed by blocking with protein block for 10 minutes. Primary antibody diluted in PBS (or isotype control) was added to the slides and incubated for 1 hour at room temperature. Primary antibodies used were L1CAM (Sigma Aldrich, St. Louis, MO) used at 0.25 ug/mL, SSEA-5 (GeneTex, Irvine, CA) used at 10 ug/mL, and ALDH1A1 (Abcam, Cambridge MA) used at 5.0 ug/mL. Slides were washed three times with PBS and incubated in polymer HRP secondary antibody for 15 minutes. After washing in PBS, slides were incubated in DAB (diaminobenzidine) for 5 minutes. Prolong Gold mounting medium (Invitrogen, Inc., Grand Island, NY) was used to coverslip the slides, and three groups of 100 cells were counted for positive cells and graphed using Prism software (Graphpad, La Jolla, CA).

Retinoblastoma, adult, and embryonic tumor tissue arrays with corresponding normal tissues were obtained from US Biomax, Inc. (Rockville, MD). Prior to immunofluorescence staining of teratoma (Applied Stem Cell, Inc, Menlo Park, CA) and tumor arrays, slides were baked for 60 minutes at 60°C, treated with xylene and alcohols, boiled in sodium citrate (pH 6.0), and blocked for one hour at room temperature in 1% BSA, 0.5% Triton-X100 in PBS. For iPSC cytospin slides, the xylene, alcohol, and sodium citrate steps were omitted. Primary antibodies in blocker (or isotype control) were added to the slides for one hour at room temperature. Slides were washed three times in PBS and then incubated in TRITC and/or FITC conjugated secondary antibody (Sigma, stock concentration listed as ranging from 3–6.5 mg/mL), diluted 1 : 400 in PBS. Slides were cover-slipped using Prolong Gold mounting medium containing DAPI (Invitrogen, Inc.) and photographed using Spot Advanced software. 

For DAB staining of tumor cell lines, three groups of 100 cells were counted for immunopositive cells and averaged and graphed using Prism software. All experiments were repeated 2-3 times for reproducibility.

## 3. Results

### 3.1. SSEA-5 and L1CAM Immunoreactivity in Human Tumors

**Figure 1:**
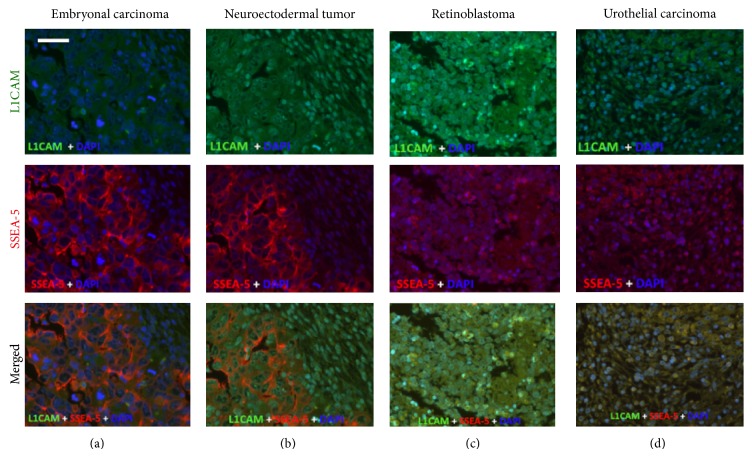
SSEA-5 and L1CAM immunoreactivity in human tumors. Tumor arrays were immunostained for both SSEA-5 (red) and L1CAM (green) as described in Section 2. For each tumor type, L1CAM, SSEA-5, and a merged image are shown. Note that the primitive tumors (embryonal carcinoma and neuroectodermal tumor) show predominantly SSEA-5 immunoreactivity (and little L1CAM immunoreactivity), while retinoblastoma shows more L1CAM immunoreactivity. The urothelial carcinoma showed immunoreactivity for both markers, with overlap in the merged image. Scale bar = 20 microns.

We evaluated immunoreactivity of SSEA-5 and L1CAM on a variety of human tumors to determine staining patterns and potential regions of colocalization. Immunofluorescence staining with SSEA-5 and L1CAM was carried out on tissue arrays of retinoblastoma, embryonic tumors, and adult tumors. As shown in Figure 1, SSEA-5 exhibited strong immunoreactivity in embryonal carcinoma and primitive neuroectodermal tumors, while L1CAM was much less intense. There were some areas of colabeling upon immunostaining with both SSEA-5 and L1CAM. Retinoblastoma displayed predominantly L1CAM staining, with some areas of SSEA-5 staining. In urothelial carcinoma, both L1CAM and SSEA-5 were seen, with many regions of colocalization. 

### 3.2. SSEA-5 and L1CAM Immunoreactivity in Human Tumor Cell Lines

**Figure 2:**
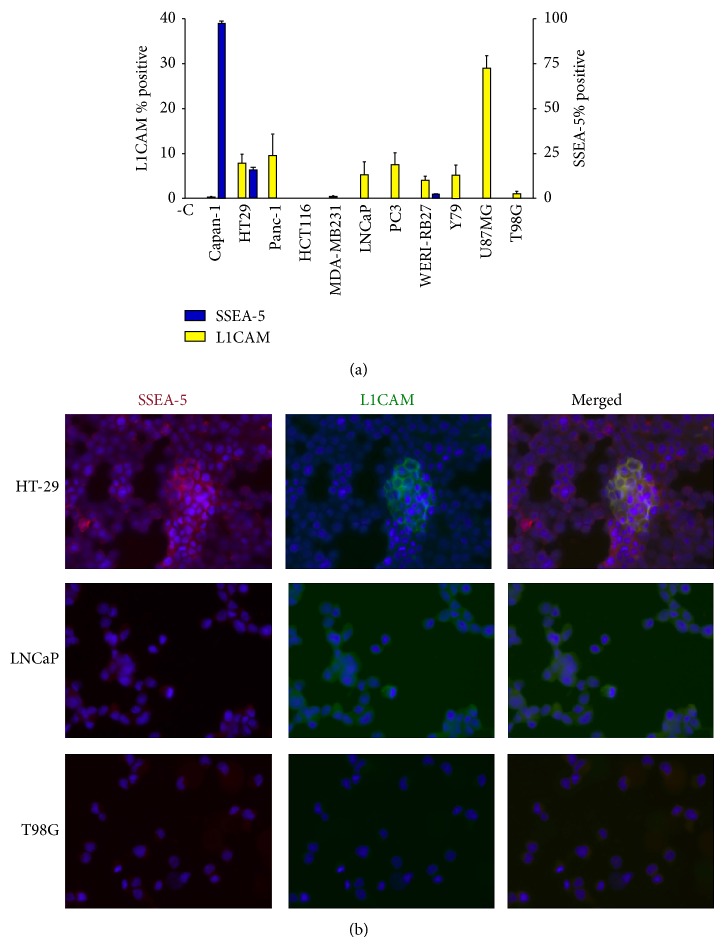
SSEA-5 and L1CAM immunoreactivity in human tumor cell lines. A variety of human tumor cell lines were immunostained with either SSEA-5 or L1CAM using diaminobenzidine reaction so that immunopositive cells could be counted in groups of 100. (a) SSEA-5 and L1CAM immunoreactivity in human tumor cells. L1CAM (yellow bars) are plotted against the left *y*-axis; SSEA-5 (blue bars) are plotted against the right *y*-axis. Capan-1 displayed the greatest number of SSEA-5+ cells, while U87MG demonstrated the highest numbers of L1CAM+ cells. For all bars not visible, the number of positive cells was less than 1%. (b) Examples of fluorescent immunoreactivity: HT-29 cells display both SSEA-5 and L1CAM immunofluorescence, with some colocalization. LNCaP cells have low levels of L1CAM immunofluorescence and no detectable SSEA-5 immunofluorescence. Microscopic fields of T98G cells are immunonegative for both L1CAM and SSEA-5.

We examined a variety of human tumor cell lines for SSEA-5 and L1CAM immunoreactivity. DAB-immunoreactive cells were counted and graphed using Prism software. L1CAM (yellow bars) are plotted against the left *y*-axis; SSEA-5 (blue bars) are plotted against the right *y*-axis. As seen in Figure 2(a), Capan-1, a pancreatic adenocarcinoma cell line, and HT-29, a colorectal adenocarcinoma cell line, showed the strongest SSEA-5 staining. Other cell lines tested were very low to immunonegative for SSEA-5. Capan-1 displayed the greatest number of SSEA-5+ cells, while U87MG demonstrated the highest numbers of L1CAM+ cells. For all bars not visible on the graph, the number of positive cells was less than 1%, as were isotype controls. In Figure 2(b), examples of immunofluorescent staining are shown. HT-29 cells demonstrated both SSEA-5 and L1CAM immunofluorescence with some colocalization. LNCaP cells have low levels of L1CAM immunofluorescence and no detectable SSEA-5 immunofluorescence. T98G cells have very few L1CAM+ cells (less than 1%) and no detectable SSEA-5.

### 3.3. SSEA-5 and L1CAM Immunoreactivity in iPS-Induced Teratoma

**Figure 3:**
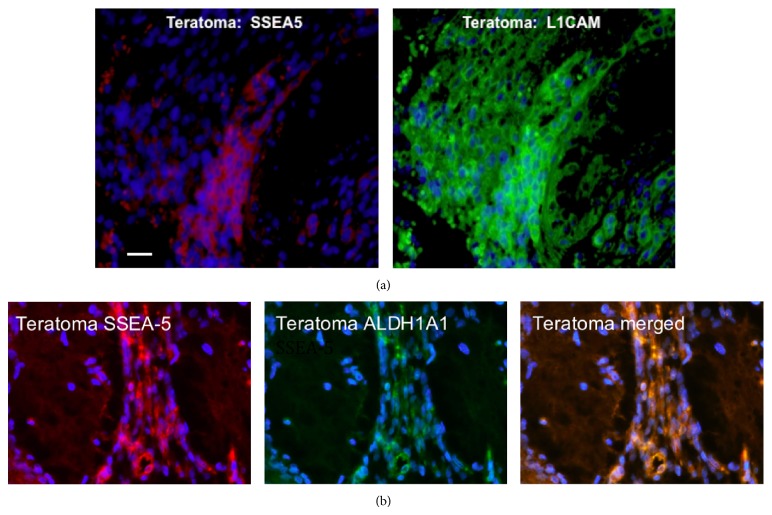
SSEA-5, L1CAM, and ALDH1A1 immunoreactivity in iPS-induced teratoma. (a) Paraffin sections of iPS-induced teratomas were obtained from Stem Cell Technologies and immunostained for both SSEA-5 (red) and L1CAM (green). The same microscopic field is shown. Note that there are some areas of colocalization for SSEA-5 and L1CAM and areas of only L1CAM immunoreactivity. Scale bar = 15 microns. (b) Colocalization of SSEA-5 with the stem cell marker ALDH1A1 is shown in iPS-induced teratoma.

Since SSEA-5 is a marker of teratoma-forming potential for iPSCs [6], we examined iPSC-induced teratomas for SSEA-5 and L1CAM immunoreactivity. Paraffin sections of teratomas grown in mice from undifferentiated iPSCs were obtained from Applied StemCell (Menlo Park, CA) and immunostained for SSEA-5 (red) and L1CAM (green). Since both SSEA-5 and L1CAM antibodies were specific for human markers, they bound specifically to human iPSCs that became part of the teratoma. In Figure 3(a), immunostaining of teratoma slides with SSEA-5 and L1CAM indicated that both of these markers were expressed, revealing the presence of SSEA-5- and L1CAM-expressing pluripotent cells. There were distinct areas of colocalization for SSEA-5 and L1CAM within the teratoma. To further validate the presence of pluripotent stem cell markers, we chose ALDH1A1, a stem cell marker [13], for a double labeling experiment with SSEA-5, seen in Figure 3(b). Colocalization of SSEA-5 and ALDH1A1 was evident in the teratoma tissue.

### 3.4. L1CAM and SSEA-5 Immunofluorescence Persists in Differentiating iPSCs

**Figure 4:**
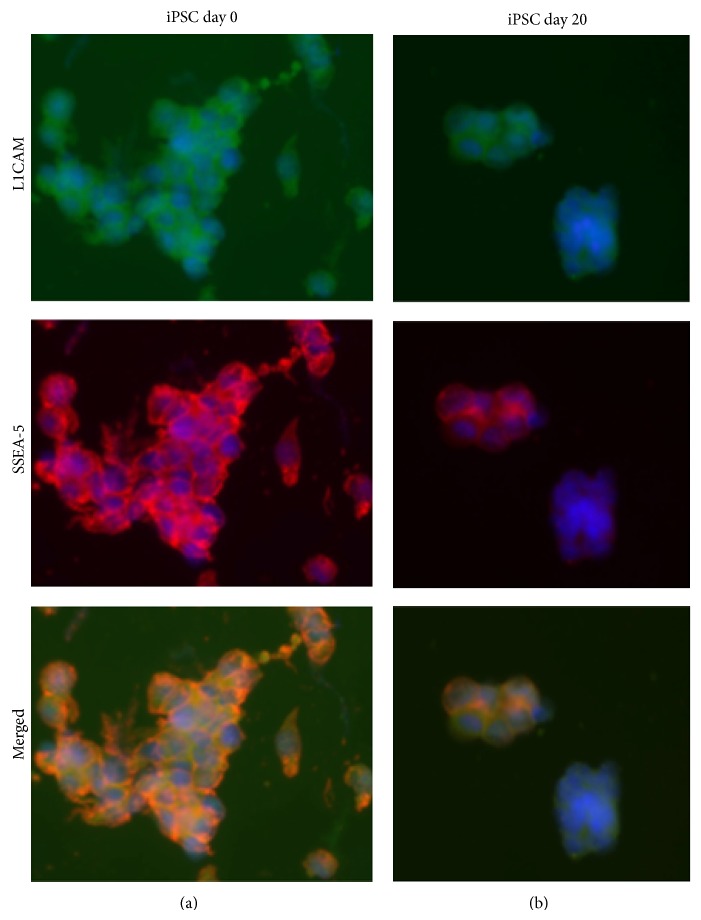
L1CAM and SSEA-5 immunofluorescence persists in differentiating iPSCs. IPSCs prior to differentiation (day 0) and after twenty days of retinal differentiation (day 20) were immunofluorescently labeled with both SSEA-5 (red) and L1CAM (green). For each condition, the same microscopic field is shown for L1CAM, SSEA-5, and a merged image. Note the colocalization of SSEA-5 and L1CAM in the merged images. In addition, dual-labeled SSEA-5 and L1CAM cells remain in the differentiated iPSC population after twenty days of retinal differentiation. Scale bar = 15 microns.

Since SSEA-5 is expressed in pluripotent iPSCs [6], as well as iPSC-induced teratomas (Figure 3), we investigated the possibility that iPSCs differentiated to a retinal progenitor cell fate would contain persistent SSEA-5+ or L1CAM+ cells. Figure 4 illustrates the presence of both L1CAM and SSEA-5 in iPSCs, with undifferentiated iPSCs (day 0) exhibiting strong immunofluorescence staining and colocalization. After 20 days of differentiation (day 20), SSEA-5 and L1CAM immunoreactive iPSCs remain, also with extensive colocalization.

## 4. Discussion

This report represents the first demonstration of SSEA-5 immunoreactivity in human tumors and tumor cell lines and partial colocalization with the cell surface marker L1CAM. 

### 4.1. SSEA-5 and L1CAM in Human Tumor Samples

SSEA-5 immunoreactivity in embryonic and neuroectodermal tumors is consistent with the pluripotent nature of the marker. Although there was some colocalization with L1CAM in human tumors, there were distinct areas of SSEA-5 immunoreactivity alone. The regions of single versus double staining for SSEA-5 and L1CAM require further analysis to determine other important regional differences within these tumors. We find biological variation in the percentage of positive cells for most markers in a variety of tumors and cell lines. The percentage of positive cells may depend on the source of the tumor (different patient samples), regional variation within a particular tumor, primitive versus differentiated state of the tumor, and so forth. So we are very careful not to overgeneralize the presence of a marker in all tumors of a particular type. Since these samples contained all tumor cells, it was not possible to identify specific tissue cell types as being immunoreactive. Additional studies are necessary with a large repository of tumors in order to determine whether SSEA-5 and/or L1CAM may be useful for diagnostic histopathology in a clinical setting.

### 4.2. SSEA-5 and L1CAM in Human Tumor Cell Lines

SSEA-5 and L1CAM displayed immunoreactivity in different subsets of tumor cell lines. The heterogeneity of these cell lines is not surprising, considering that in our previous studies, ABCG2 was expressed in 4% of retinoblastoma cell lines [10–12]. Capan-1 and HT-29, both originating from adenocarcinomas, were the only two cell lines in our study to exhibit significant SSEA-5 immunoreactivity. Interestingly, Capan-1, a pancreatic adenocarcinoma cell line, had a high percentage of SSEA-5+ cells, while Panc-1 (a pancreatic carcinoma cell line) was immunonegative for SSEA-5. At the same time, Panc-1, LNCaP, WERI-RB27, Y79, and U87MG cell lines contained L1CAM immunoreactive cells, yet none of these cell lines displayed high levels of SSEA-5 immunoreactivity. This list of cell lines is not exhaustive and further investigation into the presence these markers in a variety of tumor cell types is warranted to determine the significance of SSEA-5 and L1CAM expression. Once a sequence is established for SSEA-5, PCR primers can be designed for further analysis.

### 4.3. SSEA-5 and L1CAM in Teratomas and iPSCs

Stem cells have considerable therapeutic potential, especially with the advent of induced pluripotent stem cells (iPSCs) [14]. The potential of iPSCs as therapeutic agents for disease treatment is great but comes with the risk of uncontrolled growth of residual pluripotent cells [15]. iPSCs share common pathways with oncogenic foci [16], and there are recent reports of mutations and epigenetic changes in iPSCs that warrant further investigation [17, 18]. In this study, we demonstrated SSEA-5 and L1CAM immunoreactivity with areas of colocalization in iPSC-induced teratomas, as well as iPSCs before and after 20 days of differentiation to a retinal progenitor cell fate. Our results indicate that residual L1CAM+ and SSEA-5+ cells remain in iPSCs after 20 days of differentiation. These results confirm the importance and point to a potential means of evaluating the persistence of undifferentiated iPSCs cells following differentiation protocols. 

Future studies will reveal the utility of SSEA-5 and L1CAM in diagnostic and therapeutic strategies in both cancer biology and regenerative medicine.
